# Supplementary material for: Cu and As(V) Adsorption and Desorption on/from Different Soils and Bio-Adsorbents
Source: Materials (Basel). 2022 Jul 19;15(14):5023. doi: 10.3390/ma15145023 (PMC9323072; doi:10.3390/ma15145023)
Supplement: Supplementary file 1 [file materials-15-05023-s001.zip › materials-1777955-supplementary.pdf]

Supplementary Material

Cu and As(V) adsorption and desorption on/from different soils and bio-adsorbents

Raquel Cela-Dablanca[1*], Ana Barreiro[1], Gustavo Ferreira-Coelho[1], Claudia Campillo-Cora[2], Paula Pérez-Rodríguez[2], Manuel Arias-Estévez[2], Avelino Núñez-Delgado[1], Esperanza Álvarez-Rodríguez[1], María J. Fernández-Sanjurjo[1]

[1] Department of Soil Science and Agricultural Chemistry, Engineering Polytechnic School, Univ. Santiago de Compostela, 27002 Lugo, Spain
[2] Soil Science and Agricultural Chemistry, Fac. Sciences, Univ. Vigo, 32004 Ourense, Spain

[*] Corresponding author

Determination of BET surface area of the bio-adsorbents

To carry out the determination, ASAP 2020 equipment (MICROMERITICS) was used, providing nitrogen adsorption-desorption isotherms. Replicate quantifications were performed (n=3), with coefficients of variation always lower than 5%.

In order to assure the quality/assurance of the results, standard calibration and measurement conditions were used.

Table S1. Data corresponding to the BET surface area results for the six soils studied. Mean values (n=3) with coefficients of variation always <5%.

	**Soil**					
	**3AL**	**19AL**	**50AL**	**6S**	**51S**	**71S**
Amount of sample (g)	2.3359	2.3638	2.5429	2.1257	2.1137	2.2888
COLD (cm^3)	50.3433	46.9199	46.2303	48.2006	61.9809	44.7715
WARM (cm^3)	15.3617	15.8015	15.5919	16.1646	20.2309	15.3185
BET (m^2 g^{-1})	1.647	1.7161	2.0807	5.1653	5.4753	6.0713

Table S2. Data corresponding to the BET surface area results for the three bio-adsorbent materials studied. Mean values (n=3) with coefficients of variation always <5%.

	Oak ash	Pine bark	Mussel shell
Amount of sample (g)	1.9959	1.7630	2.9101
COLD (cm^3)	49.3853	45.6137	45.6374
WARM (cm^3)	15.6920	15.3332	15.3671
BET (m^2 g^{-1})	1.3336	0.3320	1.1318
